# Contributions to the knowledge of oribatid mites of Indonesia. 2. The genus *Pergalumna* (Galumnidae) with description of a new species and key to known species in the Oriental region (Acari, Oribatida)

**DOI:** 10.3897/zookeys.529.6421

**Published:** 2015-10-26

**Authors:** Sergey G. Ermilov, Dorothee Sandmann, Bernhard Klarner, Rahaju Widyastuti, Stefan Scheu

**Affiliations:** 1 Tyumen State University, Tyumen, Russia; 2 Georg August University Göttingen, J.F. Blumenbach Institute of Zoology and Anthropology, Göttingen, Germany; 3 Institut Pertanian Bogor, Bogor, Indonesia

**Keywords:** Oribatid mites, *Pergalumna*, new species, new record, key, Indonesia, Oriental region

## Abstract

A new species of oribatid mite of the genus *Pergalumna* (Oribatida, Galumnidae) is described from litter and soil materials in Sumatra, Indonesia. *Pergalumna paraindistincta* **sp. n.** is morphologically most similar to *Pergalumna indistincta* Ermilov & Anichkin, 2011, *Pergalumna pertrichosa* Mahunka, 1995 and *Pergalumna sura* Balogh, 1997; however, the new species differs from *Pergalumna indistincta* by the smaller body size, presence of long adanal setae *ad*_1_, and large, single median pore in females and males; from *Pergalumna pertrichosa* by the smaller body size, presence of three pairs of notogastral porose areas, elongated *A1* and minute anal setae; from *Pergalumna sura* by the presence of strong adanal setae *ad*_1_, large, single median pore in females and males, and shorter bothridial setae. Furthermore, *Pergalumna hawaiiensis hawaiiensis* (Jacot, 1934) and *Pergalumna panayensis* Ermilov & Corpuz-Raros, 2015 are recorded for the first time in the Indonesian fauna. An identification key to the known species of *Pergalumna* in the Oriental region is given.

## Introduction

This work is a part of a continuing study on the Indonesian fauna of oribatid mites, and it includes the data on the genus *Pergalumna* Grandjean, 1936 (Oribatida, Galumnidae). During taxonomic identification, four species were identified, including one new to science. The primary goal of the paper is to present data on the specific localities, notes on new records and overall known distributions of registered taxa and to describe the new species.

*Pergalumna* is a genus that was proposed by Grandjean (1936) with *Oribata nervosa* Berlese, 1914 as type species. Based on an updated generic diagnosis (Ermilov et al. 2013b), it comprises more than 140 species (Subías 2004, updated 2015; Ermilov and Bayartogtokh 2015; Ermilov and Corpuz-Raros 2015) having collectively a cosmopolitan distribution (Subías 2004, updated 2015). The identification keys to selected species were given by Shaldybina (1975), Balogh and Balogh (1990, 2002), Weigmann (2006), and Ermilov et al. (2014b). The secondary goal of the paper is to provide an identification key to known species of *Pergalumna* in the Oriental region.

## Materials and methods

Exact collection locality and habitat are given in the respective “Material examined” section for each species.

Specimens were mounted in lactic acid on temporary cavity slides for measurement and illustration. The body length was measured in lateral view, from the tip of the rostrum to the posterior edge of the ventral plate. Notogastral width refers to the maximum width in dorsal aspect. Lengths of body setae were measured in lateral aspect. All body measurements are presented in micrometers. Formulas for leg setation are given in parentheses according to the sequence trochanter–femur–genu–tibia–tarsus (famulus included). Formulas for leg solenidia are given in square brackets according to the sequence genu–tibia–tarsus.

General terminology used in this paper follows that of Grandjean (summarized by Norton and Behan-Pelletier 2009).

Drawings were made with a camera lucida using a Carl Zeiss transmission light microscope “Axioskop-2 Plus”.

## Description

### Pergalumna paraindistincta sp. n.

Taxon classificationAnimaliaOribatidaGalumnidae

http://zoobank.org/F1F876B8-867F-4EA9-851D-FB4186C05342

Figs 1 , 2 , 3–4 , 5–9

#### Diagnosis.

Body size: 415–481 × 298–365. Rostral, lamellar and interlamellar setae well developed, barbed. Bothridial setae long, setiform, ciliate. Anterior notogastral margin not developed. Three pairs of elongate oval porose areas on notogaster, *Aa* transversally oriented, located between *la* and *lm*, *A1* longitudinally oriented. Median pore single, large. Adanal setae *ad*_1_ of medium size, straight, heavily barbed. Postanal porose area absent.

#### Description.

*Measurements*. Body length: 431 (holotype: male), 415–481 (10 paratypes: three females and seven males); notogaster width: 298 (holotype), 298–365 (10 paratypes). Without sexual dimorphism.

*Integument*. Body color brown. Body surface microgranulate, visible under high magnification, ×1000 (diameter of granules less than 1).

**Figure 1.**
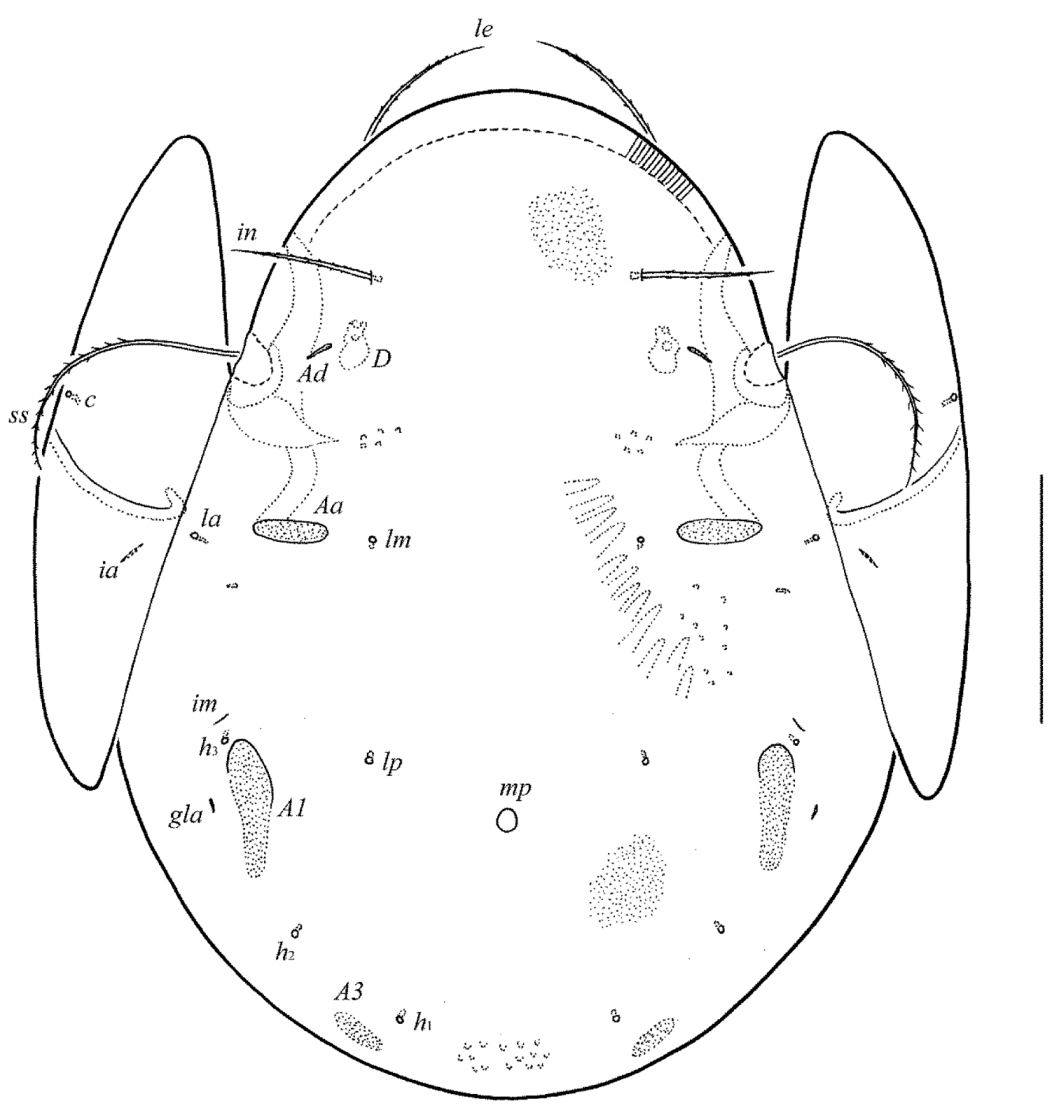
*Pergalumna paraindistincta* sp. n., adult: dorsal view. Scale bar 100 μm.

*Prodorsum* (Figs 1, 3, 5). Rostrum broadly rounded. Lamellar (*L*) and sublamellar (*S*) lines distinct, parallel, curving backwards. Rostral (*ro*, 41–49), lamellar (*le*, 69–77) and interlamellar (*in*, 86–90) setae setiform, barbed. Bothridial setae (*bs*, 114–127) setiform, densely ciliate in medio-distal parts. Exobothridial setae and their alveoli absent. Porose areas *Ad* narrowly elongate oval, transversally oriented (16–20 × 4).

**Figure 2.**
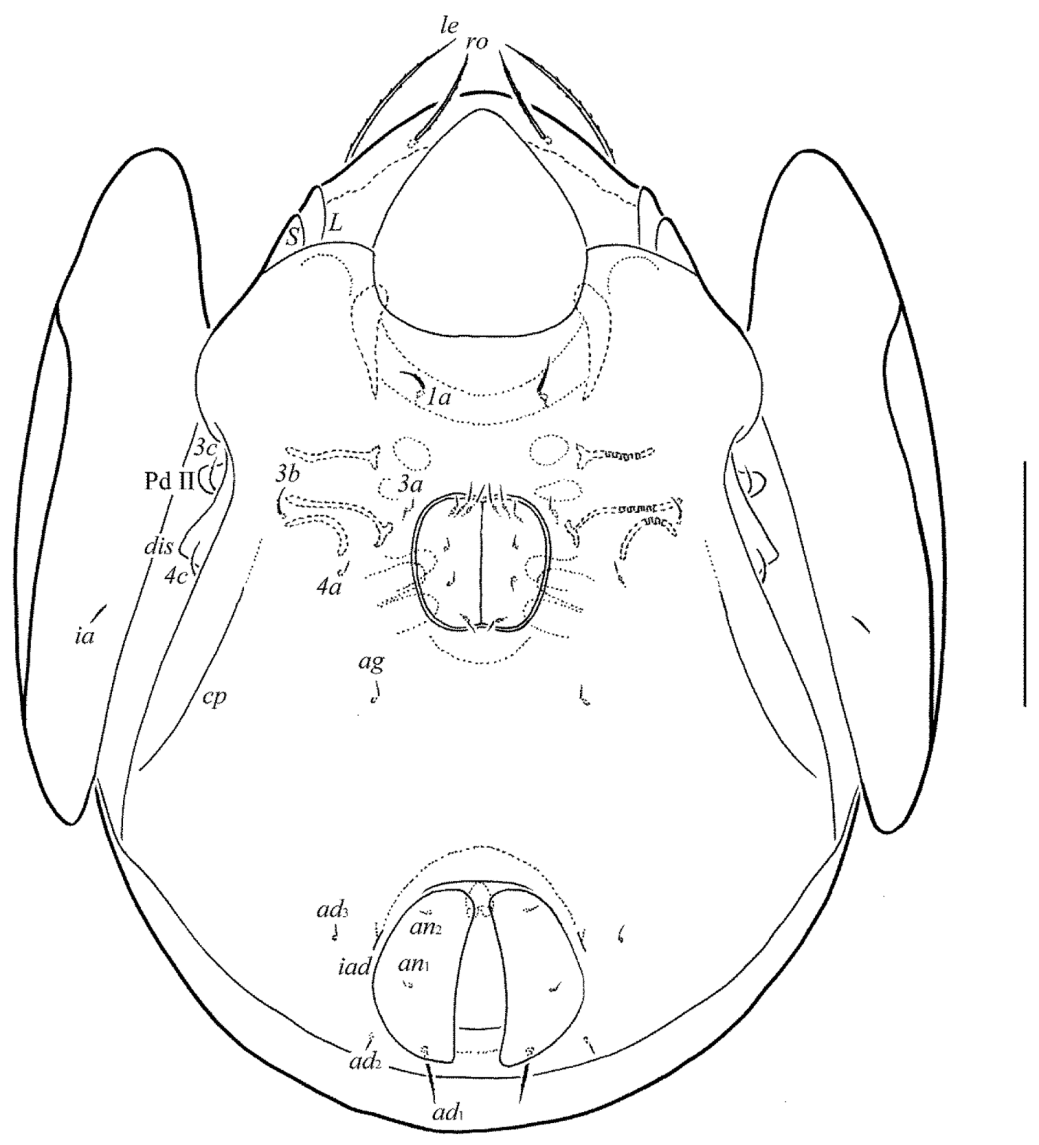
*Pergalumna paraindistincta* sp. n., adult: ventral view (gnathosoma and legs not shown). Scale bar 100 μm.

**Figures 3–4.**
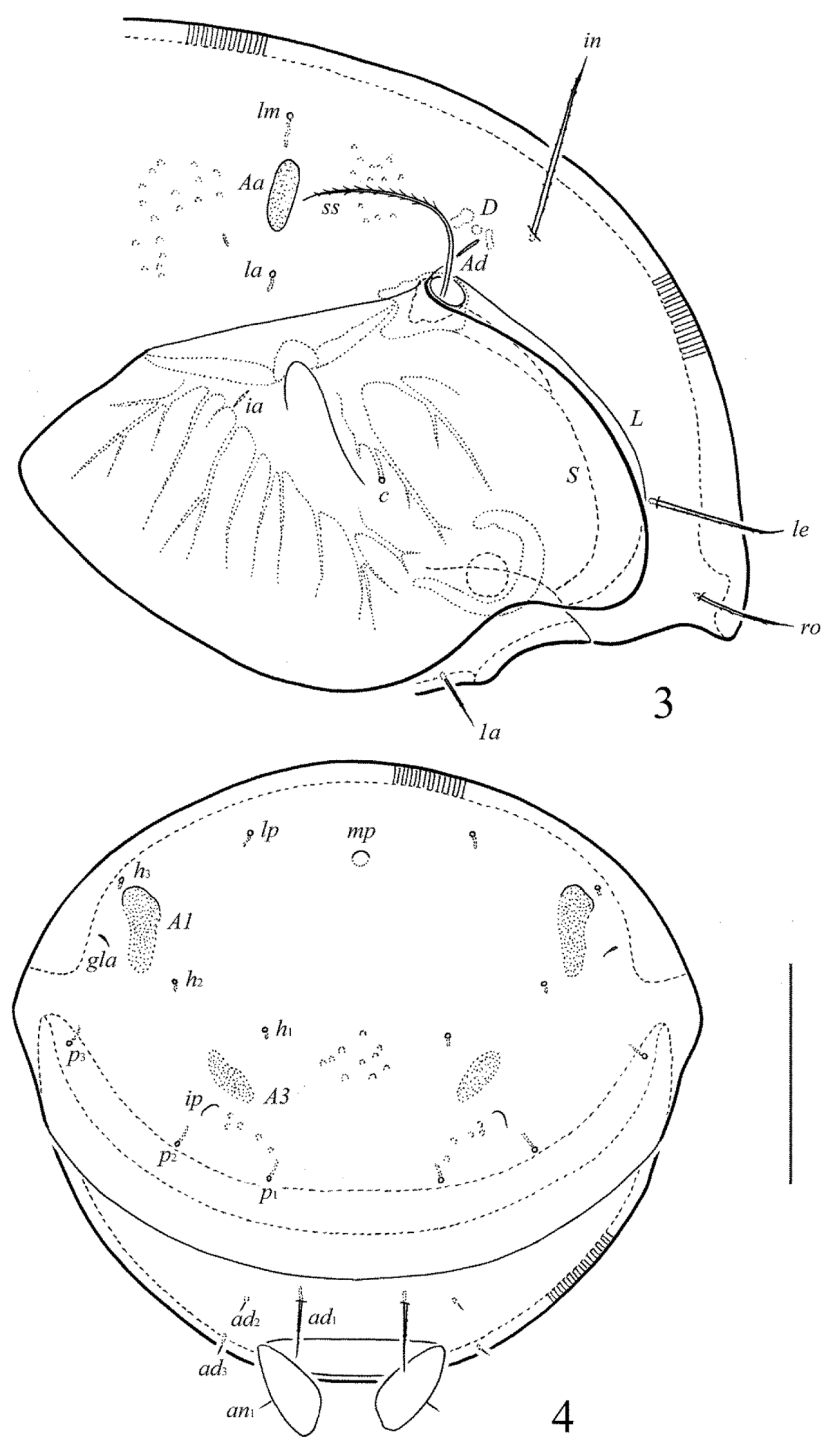
*Pergalumna paraindistincta* sp. n., adult: **3** anterior part of body, lateral view (gnathosoma and leg I not shown) **4** posterior view. Scale bar 100 μm.

**Figures 5–9.**
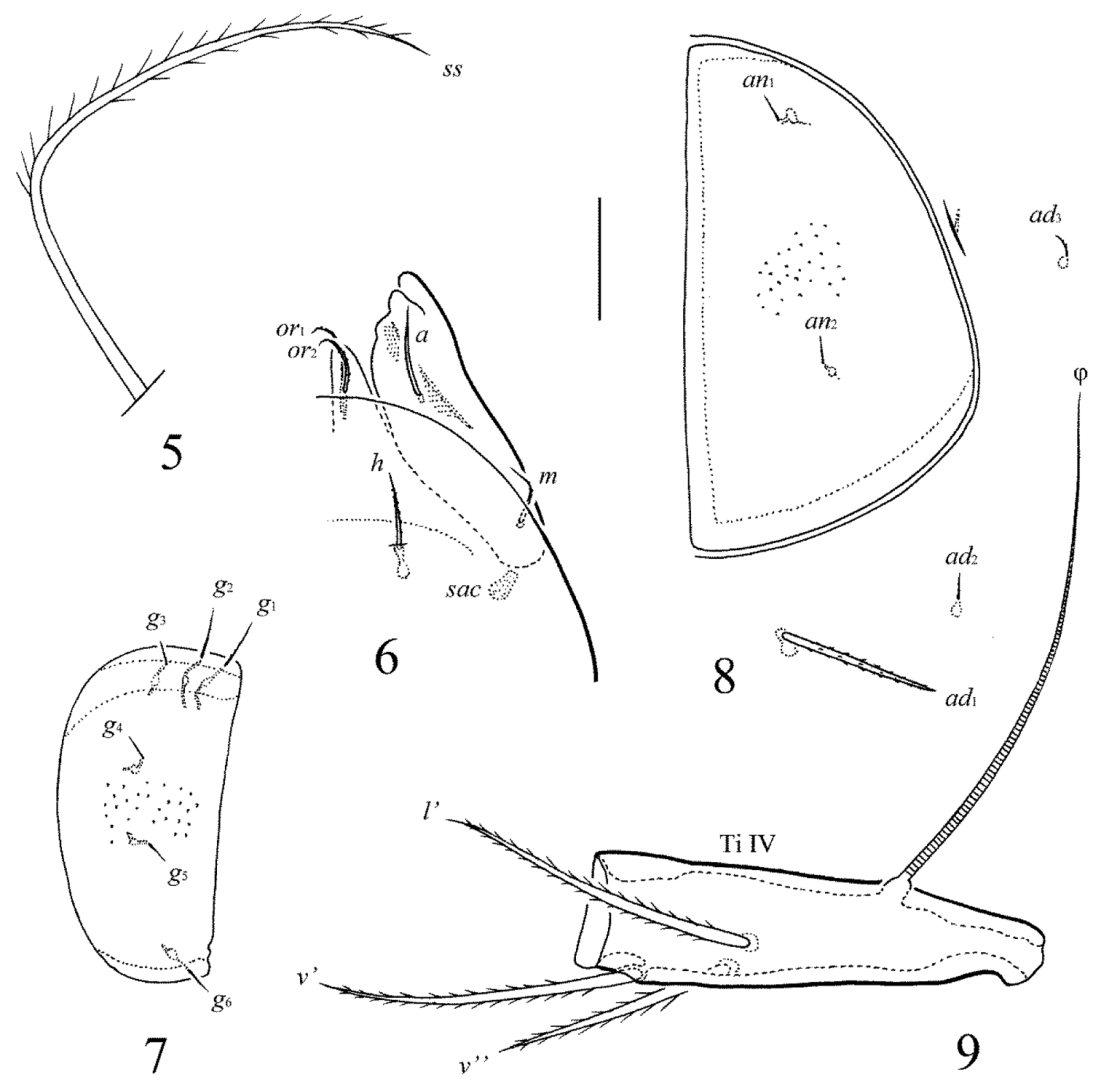
*Pergalumna paraindistincta* sp. n., adult: **5** bothridial seta **6** anterior part of left half of subcapitulum, ventral view **7** genital plate, right **8** anal plate, left, and adanal setae **9** tibia of leg IV, right, antiaxial view. Scale bar 20 μm.

*Notogaster* (Figs 1, 3, 4). Anterior notogastral margin not developed. Dorsophragmata (*D*) of medium size, elongated longitudinally. Notogastral setae represented by 10 pairs of alveoli. Three pairs of porose areas: *Aa* clearly bordered, elongate oval, transversally oriented (28–32 × 8–12), *A1* distinctly bordered only in anterior part, elongate oval to slightly elongate triangular, longitudinally oriented (57–68 × 12–16), *A3* without clear borders, elongate oval (24–28 × 8–12). Areas *Aa* located between setal alveoli *la* and *lm*, equal distanced from them. Median pore present in males and females, comparatively large (diameter 10–16). All lyrifissures (*ia*, *im*, *ip*, *ih*, *ips*) distinct, *im* located antero-laterally to *A1*. Opisthonotal gland openings (*gla*) located laterally to *A1*.

*Gnathosoma* (Fig. 6). Morphology of subcapitulum, palps and chelicerae typical for *Pergalumna* (see Engelbrecht 1972; Ermilov and Anichkin 2011a, b). Subcapitulum size: 102–106 × 102–106. Subcapitular setae setiform, slightly barbed, *m* (14–16) shorter than *a* and *h* (both pairs 18–20); *a* thickest, *m* thinnest. Two pairs of adoral setae (*or*_1_, *or*_2_, 12–14) setiform, hook-like distally, barbed. Palps (90–94) with typical setation: 0–2–1–3–9(+ω). Axillary sacculi (*sac*) distinct. Chelicerae (164) with two setiform, barbed setae; *cha* (41) longer than *chb* (24). Trägårdh’s organ long, tapered.

*Epimeral and lateral podosomal regions* (Fig. 2). Anterior tectum of epimere I smooth. Apodemes 1, 2, sejugal and 3 well visible. Six pairs of setae, setal formula: 1–0–2–3. Setae thin, slightly barbed, *1a*, *3c* and *4c* (24) longer than *3b* (10–14) and *4a* and *4b* (4); *1a* thicker than others. Pedotecta II (Pd II) scale-like in lateral view, rounded distally in ventral view. Discidia (*dis*) sharply triangular. Circumpedal carinae (*cp*) slightly developed.

*Anogenital region* (Figs 2, 4, 7, 8). Six pairs of genital (*g*_1_, *g*_2_, 12; *g*_2_–*g*_6_, 8), one pair of aggenital (*ag*, 8), two pairs of anal (*an*_1_, *an*_2_, 8) and two pairs of adanal setae (*ad*_2_, *ad*_3_, 8) thin, indistinctly barbed. One pair of adanal setae (*ad*_1_, 24–36) thickened, straight, heavily barbed, however, in three paratypes one seta of the *ad*_1_ pair is short, as *ad*_2_ and *ad*_3_ in morphology. Adanal setae *ad*_3 _inserted laterally to adanal lyrifissures (*iad*). Genital plates with two or three setae on anterior edge of each plate. Postanal porose area absent.

*Legs* (Fig. 9). Morphology of leg segments, setae and solenidia typical for *Pergalumna* (see Engelbrecht 1972; Ermilov and Anichkin 2011a, b). Claws smooth. Formulas of leg setation and solenidia: I (1–4–3–4–20) [1–2–2], II (1–4–3–4–15) [1–1–2], III (1–2–1–3–15) [1–1–0], IV (1–2–2–3–12) [0–1–0]; homology of setae and solenidia indicated in Table 1. Solenidion φ of tibiae IV inserted dorsally in posterior part of segments.

**Table 1.** Leg setation and solenidia of adult *Pergalumna paraindistincta* sp. n.

Leg	Tr	Fe	Ge	Ti	Ta
I	*v*’	*d*, (*l*), *bv*’’	(*l*), *v*’, σ	(*l*), (*v*),﻿ φ_1_, φ_2_	(*ft*), (*tc*), (*it*), (*p*), (*u*), (*a*), *s*, (*pv*), *v*’, (*pl*), *l*’’, ε, ω_1_, ω_2_
II	*v*’	*d*, (*l*), *bv*’’	(*l*), *v*’, σ	(*l*), (*v*), φ	(*ft*), (*tc*), (*it*), (*p*), (*u*), (*a*), *s*, (*pv*), ω_1_, ω_2_
III	*v*’	*d*, *ev*’	*l*’, σ	*l*’, (*v*), φ	(*ft*), (*tc*), (*it*), (*p*), (*u*), (*a*), *s*, (*pv*)﻿
IV	*v*’	*d*, *ev*’	*d*, *l*’	*l*’, (*v*), φ	*ft*’’, (*tc*), (*p*), (*u*), (*a*), *s*, (*pv*)﻿

Note: Roman letters refer to normal setae, Greek letters to solenidia (except ε = famulus). Single prime (‘) marks setae on the anterior and double prime (“) setae on the posterior side of a given leg segment. Parentheses refer to a pair of setae. Tr – trochanter , Fe – femur , Ge – genu , Ti – Tibia , Ta – tarsus .

#### Material examined.

Holotype (male): Indonesia, Sumatra, Harapan landscape, secondary rainforest, research site HF1, 02°09'09.9"S, 103°21'43.2"E, 76 m a.s.l., from forest floor litter material. Six paratypes (two females and four males): Indonesia, Sumatra, Harapan landscape, rubber plantation, research site HR2, 01°52'44.5"S, 103°16'28.4"E, 59 m a.s.l., from forest floor litter material. Four paratypes (one female and three males): Sumatra, Indonesia, Harapan landscape, jungle rubber agroforest, research site HJ1, 01°55'40.0"S, 103°15'33.8"E, 51 m a.s.l., from forest floor litter material. All specimens were collected by Bernhard Klarner (15.XI.2013) and identified and collected to morphospecies level by Dorothee Sandmann.

#### Type deposition.

The holotype is deposited in LIPI (Indonesian Institute of Science) Cibinong, Indonesia; three paratypes are in the collection of the Senckenberg Museum, Görlitz, Germany; seven paratypes are in the collection of the Tyumen State University Museum of Zoology, Tyumen, Russia.

#### Etymology.

The specific name *paraindistincta* refers to the morphological similarity of the new species to *Pergalumna indistincta* Ermilov & Anichkin, 2011.

#### Remarks.

*Pergalumna paraindistincta* sp. n. is morphologically most similar to *Pergalumna indistincta* Ermilov & Anichkin, 2011 from Vietnam (see Ermilov and Anichkin 2011b) and *Pergalumna sura* Balogh, 1997 from the Neotropical region (see Balogh 1997; Ermilov et al. 2014a) in having rounded rostrum, well-developed prodorsal setae, setiform and ciliate of bothridial setae, three pairs of notogastral porose areas, transversally oriented *Aa* and strongly elongated, longitudinally oriented *A1*, and the absence of anterior notogastral margin as well as postanal porose area. However, the new species differs from both by the presence of strong adanal setae *ad*_1_ (vs. minute in *Pergalumna indistincta* and *Pergalumna sura*) and large, single median pore in females and males (vs. median pore absent in males and represented by several foveae in females in *Pergalumna indistincta*, and absent in females and males in *Pergalumna sura*). Additionally, the new species is smaller than *Pergalumna indistincta* (415–481 × 298–365 vs. 547–614 × 381–415) and has shorter bothridial setae than *Pergalumna sura*.

Furthermore, *Pergalumna paraindistincta* sp. n. is morphologically similar to *Pergalumna pertrichosa* Mahunka, 1995 from Borneo (see Mahunka 1995) in having a rounded rostrum, well developed prodorsal setae, setiform and ciliate bothridial setae, strong adanal setae *ad*_1_, a single median pore, and the absence of an anterior notogastral margin. However, the new species differs from the latter by the smaller body size (415–481 × 298–365 vs. 550–608 × 413–454 in *Pergalumna pertrichosa*), presence of three pairs of notogastral porose areas with elongated *A1* (vs. four pairs of porose areas with *A1* rounded in *Pergalumna pertrichosa*) and minute anal setae (vs. well developed in *Pergalumna pertrichosa*).

### Records

*Pergalumna hawaiiensis hawaiiensis* (Jacot, 1934) (see Jacot 1934a). Distribution: Pacific Islands. New record for Indonesia.

**Material examined.** One specimen: Indonesia, Sumatra, Harapan landscape, Jungle rubber agroforest, research site HJ1, 01°55'40.0"S, 103°15'33.8"E, 51 m a.s.l., from upper soil layer (0–5 cm), 15.XI.2013 (B. Klarner). Three specimens: Indonesia, Sumatra, Bukit Duabelas landscape, rubber plantation, research site BR2, 02°05'06.8"S, 102°47'20.7"E, 95 m a.s.l., from upper soil layer (0–5 cm), 15.XI.2013 (B. Klarner). One specimen: Indonesia, Sumatra, Bukit Duabelas landscape, oil palm plantation, research site BO3, 02°04'15.2"S, 102°47'30.6"E, 71 m a.s.l., from upper soil layer (0–5 cm), 15.XI.2013 (B. Klarner).

*Pergalumna panayensis* Ermilov & Corpuz-Raros, 2015 (see Ermilov and Corpuz-Raros 2015). Distribution: Philippines. New record for Indonesia.

**Material examined.** One specimen: Indonesia, Sumatra, Harapan landscape, rubber plantation, research site HR2, 01°52'44.5"S, 103°16'28.4"E, 59 m a.s.l., from forest floor litter material, 15.XI.2013 (B. Klarner). One specimen: same data, but in upper soil layer (0–5 cm). One specimen: Indonesia, Sumatra, Bukit Duabelas landscape, jungle rubber agroforest, research site BJ4, 02°00'57.3"S, 102°45'12.3"E, 60 m a.s.l., from upper soil layer (0–5 cm), 15.XI.2013 (B. Klarner).

*Pergalumna pterinervis* (Canestrini, 1898) (see Mahunka 1992). Distribution: Oriental region. New record for Indonesia.

**Material examined.** One specimen: Indonesia, Sumatra, Harapan landscape, jungle rubber agroforest, research site HJ2, 01°49'31.9’S’, 103°17'39.2"E, 84 m a.s.l., from forest floor litter material, 15.XI.2013 (B. Klarner). One specimen: Indonesia, Sumatra, Harapan landscape, jungle rubber, research site HJ4, 01°47'07.3"S, 103°16'36.9"E, 57 m a.s.l., from upper soil layer (0–5 cm), 15.XI.2013 (B. Klarner). One specimen: Indonesia, Sumatra, Bukit Duabelas landscape, secondary rainforest, research site BF2, 01° 58'55.1"S, 102°45'02.7"E, 77 m a.s.l., from upper soil layer (0–5 cm), 15.11.2013 (B. Klarner). Three specimens: Indonesia, Sumatra, Harapan landscape, jungle rubber agroforest, research site HJ2, 01°49'31.9"S, 03°17'39.2"E, 84 m a.s.l., from forest floor litter material, 15.XI.2013 (B. Klarner).

### Key to known species of *Pergalumna* in the Oriental region

At present, 45 species/subspecies of *Pergalumna* are known in the Oriental region (Subías 2004, updated 2015; including present and personal data of the first author).

*Pergalumna heroica* (Willmann, 1931) from Java (see Willmann 1931), *Pergalumna medialis* (Sellnick, 1925) from Sumatra (see Sellnick 1925) and *Pergalumna obsessa* Subías, 2004 from Taiwan (see Tseng 1984 as *Galumna pallida* Tseng, 1984) are excluded from the key because these species have been poorly described.

*Pergalumna curva curva* (Ewing, 1907) from the Holarctic and Oriental regions (see Ewing 1907; Jacot 1934b), *Pergalumna curva ventralis* (Willmann, 1931) from the Holarctic, Neotropical and Oriental regions and Polynesia (see Willmann 1931; Jacot 1934b; Hammer 1958, 1961, 1972), *Pergalumna obvia obvia* (Berlese, 1914) from the Ethiopian, Neotropical, Oriental and Palaearctic regions, and Hawaii and U.S.A. (see Weigmann 2006; Ermilov et al. 2013c), *Pergalumna operata* Tseng, 1984 from Taiwan (see Tseng 1984) and *Pergalumna pyramidalis* (Tseng, 1984) from Taiwan (see Tseng 1984) were not included because systematic placement of these species is not clear. We consider *Pergalumna obvia obvia* as a representative of the genus *Galumna* Heyden, 1826 (see Ermilov et al. 2013c). *Pergalumna curva curva*, *Pergalumna curva ventralis* and *Pergalumna operata* Tseng, 1984 have distinct notogastral setae, which are not traits for *Pergalumna* (see generic diagnosis in Ermilov et al. 2013b); these species are poorly described and redescribed; however, based on available data they should be considered as representatives of the genus *Allogalumna* Grandjean, 1936 or *Trichogalumna* Balogh, 1960 (depending on presence or absence of lamellar lines). *Pergalumna pyramidalis* has 14 pairs of notogastral setal alveoli, developed lamellae directed to insertions of lamellar setae and pteromorphs without setae (Tseng 1984); most likely this species is a representative of the subgenus Neoribates (Neoribates) Berlese, 1914 (Oripodoidea, Parakalummidae), and it is morphologically most similar to Neoribates (Neoribates) paratuberculatus Ermilov, Shtanchaeva & Subías, 2014 from Vietnam (see Ermilov et al. 2014d) and Neoribates (Neoribates) tuberculatus Willmann, 1956 from “Czechoslovakia” (see Willmann 1956) in having tubercles on pteromorphs and some other characters.

**Table:** 

1	Anterior margin of notogaster of specific structure, tuberculate	**2**
–	Anterior margin of notogaster simple, smooth or not developed	**3**
2	Genital plates with several striae; notogastral porose areas of medium size, larger than diameter of bothridia; body size: 451–490 × 328–366	***Pergalumna margaritata* Mahunka, 1989 (Mahunka 1989)**. Distribution: Vietnam
–	Genital plates with one pair of striae; notogastral porose areas small, similar to diameter of bothridia; body size: 402–447 × 281–315	***Pergalumna pseudomargaritata* Mahunka, 1994** (see Mahunka 1994). Distribution: Thailand
3	Anterior margin of notogaster distinctly developed, complete	**4**
–	Anterior margin of notogaster not developed	**12**
4	Rostrum pointed	**5**
–	Rostrum rounded	**7**
5	Four pairs of notogastral porose areas; *Aa* elongate triangular, transversally oriented; lateral parts of pteromorphs with strong ridges forming slightly visible reticulate pattern; body size: 517–670 × 397–525	***Pergalumna altera* (Oudemans, 1915)** (see Aoki 1961 as *Pergalumna harunaensis* Aoki 1961, 1975; Engelbrecht 1972; Weigmann 2006). Distribution: Semicosmopolitan
–	Three pairs of notogastral porose areas; *Aa* rounded; pteromorphs without strong ridges and reticulate pattern	**6**
6	Interlamellar setae long; posterior part of notogaster without furrows; body size: 664–830 × 498–630	***Pergalumna yurtaevi* Ermilov & Anichkin, 2011** (see Ermilov and Anichkin 2011a; Ermilov et al. 2012a). Distribution: Vietnam
–	Interlamellar setae represented by alveoli; posterior part of notogaster with two parallel, longitudinal furrows; body size: 664–830 × 498–630	***Pergalumna asetosa* Ermilov, Shtanchaeva, Kalúz & Subías, 2013** (see Ermilov et al. 2013a). Distribution: India
7	Bothridial setae setiform; body size: 520–676 × 502	***Pergalumna foveolata* Hammer, 1973** (see Hammer 1973; Bayartogtokh and Chatterjee 2010). Distribution: Australian, Neotropical and Oriental region
–	Bothridial setae with developed head	**8**
8	Interlamellar setae minute; body surface foveolate; body size: 222–235 × 177–190	***Pergalumna annulata* Mahunka, 1995** (see Mahunka 1995). Distribution: Borneo
–	Interlamellar setae long; body surface not foveolate	**9**
9	Three pairs of notogastral porose areas; *Aa* rounded; body length: 820	***Pergalumna corniculata* (Berlese, 1905)** (see Berlese 1905; Mahunka 1992). Distribution: Java
–	Four pairs of notogastral porose areas; *Aa* elongated, transversally oriented	**10**
10	Notogastral porose areas *Aa* triangular; median pore present; body size: 623 × 533	***Pergalumna taprobanica* Balogh, 1988** (see Balogh 1988). Distribution: Oriental region
–	Notogastral porose areas *Aa* elongate oval to boot-shaped	11
11	Bothridial setae fusiform, with well-developed head rounded distally; postanal porose area present; body size: 672 × 528	***Pergalumna andhraense* Raju, Appalanaidu & Rao, 1981** (see Raju et al. 1981). Distribution: India
–	Bothridial setae lanceolate, with slightly developed head pointed distally; postanal porose area absent; body size: 830–898 × 630–680	***Pergalumna paraelongata* Ermilov & Anichkin, 2012** (see Ermilov et al. 2012b). Distribution: Vietnam
12	Rostrum trapezoid; anal setae comparatively long, longer than width of anal plate; body size: 1278–1311 × 976–1045	***Pergalumna paraclericata* Ermilov, Chatterjee, Das & Bordoloi, 2014** (see Ermilov et al. 2014c). Distribution: India
–	Rostrum not trapezoid; anal setae comparatively short, shorter than width of anal plate	**13**
13	Rostrum pointed	**14**
–	Rostrum rounded	**18**
14	Four pairs of notogastral porose areas; *Aa* located nearer to setal alveoli *la* than *lm*; body size: 730–780 × 564–597	***Pergalumna cattienica* Ermilov & Anichkin, 2011** (see Ermilov and Anichkin 2011a). Distribution: Vietnam
–	Three pairs of notogastral porose areas; *Aa* located nearer to setal alveoli *lm* or distanced equal from *la* and *lm*	**15**
15	Interlamellar setae represented by alveoli; anterior part of prodorsum with two longitudinal ridges; notogastral porose areas *Aa* located nearer to setal alveoli *lm* than *la*; body size: 1162–1278 × 898–1012	***Pergalumna minipora* Ermilov, Chatterjee, Das & Bordoloi, 2014** (see Ermilov et al. 2014c). Distribution: India
–	Interlamellar setae of medium size or long; prodorsum without ridges; notogastral porose areas *Aa* distanced equal from *la* and *lm*	**16**
16	Notogastral porose areas *A1* elongated, longitudinally oriented; body surface foveolate; genital plates not striate; body size: 365–415 × 265–332	***Pergalumna paratsurusakii* Ermilov, Shtanchaeva, Kalúz & Subías, 2013** (see Ermilov et al. 2013a). Distribution: India
–	Notogastral porose areas *A1* rounded; body surface not foveolate; genital plates striate	**17**
17	Adanal setae *ad*_1_ and *ad*_2_ comparatively long, not shorter than width of anal plate; median pore absent; interlamellar setae longer than bothridial setae; body size: 597–680 × 431–498	***Pergalumna paracattienica* Ermilov, Chatterjee, Das & Bordoloi, 2014** (see Ermilov et al. 2014c). Distribution: India
–	Adanal setae *ad*_1_ and *ad*_2_ minute; median pore present; interlamellar setae shorter than bothridial setae; body size: 498–531 × 381–398	***Pergalumna mahunkai* Ermilov, Shtanchaeva, Kalúz & Subías, 2013** (see Ermilov et al. 2013a). Distribution: India
18	Four pairs of notogastral porose areas	**19**
–	Three pairs of notogastral porose areas	**22**
19	Interlamellar setae represented by alveoli; notogastral porose areas *Aa* located anteriorly to setal alveoli *la*; body length: 730	***Pergalumna corolevuensis* Hammer, 1973** (see Hammer 1973). Distribution: Fiji and India
–	Interlamellar setae of medium size or long; notogastral porose areas *Aa* located between setal alveoli *la* and *lm*	**20**
20	Notogastral porose areas *A1* located antero-medially to *A2*; interlamellar setae of medium size; body size: 745–842 × 567–640	***Pergalumna hauseri* Mahunka, 1995** (see Mahunka 1995). Distribution: Borneo
–	Notogastral porose areas *A1* located anteriorly to *A2*; interlamellar setae long	**21**
21	Adanal setae *ad*_1_ and *ad*_2_ similar in length; median pore absent; body size: 510–630 × 410–481	***Pergalumna pterinervis* (Canestrini, 1898)** (see Canestrini 1898; Berlese 1905, 1914; Mahunka 1992; including our data). Distribution: Oriental region
–	Adanal setae *ad*_1_ longer than *ad*_2_; median pore present; body size: 550–608 × 413–454	***Pergalumna pertrichosa* Mahunka, 1995** (see Mahunka 1995). Distribution: Borneo
22	Notogastral porose areas *Aa* located nearer to setal alveoli *la* than *lm*; bothridial setae clavate	**23**
–	Notogastral porose areas *Aa* located nearer to setal alveoli *lm* than *la* or distanced equal from them; bothridial setae setiform or with slightly dilated, elongate head	**24**
23	Interlamellar setae minute, shorter than diameter of bothridia; body surface not foveolate; median pore represented by several foveae; body size: 262–282 × 192–209	***Pergalumna pseudosejugalis* Ermilov & Anichkin, 2012** (see Ermilov and Anichkin 2012). Distribution: Vietnam
–	Interlamellar setae short, but longer than diameter of bothridia; body surface foveolate; median pore absent; body size: 246–275 × 186–212	***Pergalumna crassipora* Mahunka, 1995** (see Mahunka 1995). Distribution: Borneo
24	Notogastral porose areas *Aa* located nearer to setal alveoli *lm* than *la*	**25**
–	Notogastral porose areas *Aa* distanced equal from *la* and *lm*	**30**
25	Notogastral porose areas minute, smaller than diameter of bothridia; body size: 527–612 × 390–428	***Pergalumna imadatei* Aoki & Hu, 1993** (see Aoki and Hu 1993). Distribution: Oriental region
–	Notogastral porose areas well developed, larger than diameter of bothridia	**26**
26	Body surface slightly striate; median pore represented by several foveae; body size: 610–715 × 475–545	***Pergalumna hawaiiensis hawaiiensis* (Jacot, 1934)** (see Jacot 1934a; including our data)
–	Body surface not striate; median pore single or absent	**27**
27	Interlamellar setae minute, shorter than diameter of bothridia; body length: 720	***Pergalumna bimaculata* Hammer, 1973** (see Hammer 1973). Distribution: Polynesia and Philippines
–	Interlamellar setae of medium size, longer than diameter of bothridia	**28**
28	Median pore present, large; body length: 720	***Pergalumna remota* (Hammer, 1968)** (see Hammer 1968). Distribution: New Zealand and India
–	Median pore absent	**29**
29	Bothridial setae densely ciliate; body size: 451–490 × 300–334	***Pergalumna kotschyi* Mahunka, 1989** (see Mahunka 1989). Distribution: Vietnam
–	Bothridial setae smooth; body size: 398–453 × 275–340	***Pergalumna indivisa* Mahunka, 1995** (see Mahunka 1995). Distribution: Borneo
30	Bothridial setae with slightly dilated, elongated head	**31**
–	Bothridial setae setiform	**33**
31	Body surface heavily tuberculate; body size: 385–425 × 285–331	***Pergalumna granulata* Balogh & Mahunka, 1967** (see Balogh and Mahunka 1967). Distribution: Vietnam and Japan
–	Body surface not tuberculate	**32**
32	Body surface heavily granulate; body size: 302–356 × 237–262	***Pergalumna punctulata* Balogh & Mahunka, 1967** (see Balogh and Mahunka 1967). Distribution: Vietnam
–	Body surface smooth; body size: 437–465 × 310–324	***Pergalumna intermedia intermedia* Aoki, 1963** (see Aoki 1963, 1966). Distribution: Palaearctic and Oriental regions
33	Notogastral porose areas *A1* elongated, longitudinally oriented	**34**
–	Notogastral porose areas *A1* rounded to oval	**36**
34	Adanal setae *ad*_1_ long, not shorter than width of anal plate; median pore single; body size: 415–481 × 298–365	***Pergalumna paraindistincta* sp. n.** Distribution: Indonesia
–	Adanal setae *ad*_1_ minute; median pore absent or represented by several foveae	**35**
35	Postanal porose area absent; median more present in females; body size: 547–614 × 381–415	***Pergalumna indistincta* Ermilov & Anichkin, 2011** (see Ermilov and Anichkin 2011b). Distribution: Vietnam
–	Postanal porose area present; median more absent in females; body size: 576 × 426	***Pergalumna magnipora capensis* Engelbrecht, 1972** (see Engelbrecht 1972). Distribution: Southern Africa and India
36	Body surface striate and short ridges; posterior part of notogaster with longitudinal concavity; body size: 408–485 × 298–352	***Pergalumna menglunensis* Aoki & Hu, 1993** (see Aoki and Hu 1993). Distribution: southern China
–	Body not striate and without short ridges; posterior part of notogaster without concavity	**37**
37	Adanal setae *ad*_1_ and *ad*_2_ comparatively long, not shorter than width of anal plate; setae *c* developed on pteromorphs; body size: 514–597 × 365–431	***Pergalumna minituberculata* Ermilov & Martens, 2014** (see Ermilov and Martens 2014). Distribution: Nepal
–	Adanal setae *ad*_1_ and *ad*_2_ shorter than width of anal plate; setae *c* represented by alveoli on pteromorphs	**38**
38	Interlamellar setae represented by alveoli; median pore present; body size: 863–1145 × 639–970	***Pergalumna panayensis* Ermilov & Corpuz-Raros, 2015** (see Ermilov and Corpuz-Raros 2015)
–	Interlamellar setae of medium size or long; median pore absent	**39**
39	Bothridial setae densely ciliate	**40**
–	Bothridial setae densely smooth	**41**
40	Notogastral porose areas amorphous, without distinct borders; genital plates not striate; body size: 332–377 × 245–276	***Pergalumna amorpha* Mahunka, 2008** (see Mahunka 2008). Distribution: Thailand
–	Notogastral porose areas with distinct borders; genital plates striate; body size: 390–435 × 282–315	***Pergalumna intermedia retroversa* Aoki & Hu, 1993** (see Aoki and Hu 1993). Distribution: southern China
41	Interlamellar setae comparatively short, about 1/3 as long as their mutual distance; genital plates smooth; body size: 742–845 × 589–653	***Pergalumna magnipora capillaris* Aoki, 1961** (see Aoki 1961). Distribution: Palaearctic and Oriental regions
–	Interlamellar setae of medium size, about 1/2 as long as their mutual distance; genital plates striate; body size: 822–840 × 618–650	***Pergalumna magnipora xishuangbanna* Aoki & Hu, 1993** (see Aoki and Hu 1993). Distribution: southern China

## Supplementary Material

XML Treatment for Pergalumna paraindistincta
